# Left hand abscess as a paradoxical reaction during treatment of disseminated tuberculosis in immunocompetent patient: case report and review of literature

**DOI:** 10.1186/s12879-024-10077-w

**Published:** 2024-10-21

**Authors:** Aisha Alharbi, Aseel Aljahdali, Mohamed Firoze Ahamed, Hassan Almarhabi

**Affiliations:** 1 Pharmaceutical Care Department, King Abdulaziz Medical City, PO Box 9515, Jeddah, 21423 Saudi Arabia; 2 King Abdullah International Medical Research Center, Jeddah, Saudi Arabia; 3 King Saud Bin Abdulaziz University for Health Sciences, Jeddah, Saudi Arabia; 4 Internal Medicine Residency Program, King Abdulaziz Medical City, Jeddah, Saudi Arabia; 5 Consultant Radiologist, Medical Imaging Department, King Abdulaziz Medical City, Jeddah, Saudi Arabia; 6 Department of Medicine, King Abdulaziz Medical City, Ministry of National Guard Health Affairs, Jeddah, Saudi Arabia

**Keywords:** Tuberculosis, TB, Paradoxical reaction

## Abstract

A paradoxical reaction (PR) during the treatment of tuberculosis was defined as the worsening of preexisting disease either clinically or radiologically or the appearance of a new tuberculous lesion. These reactions are frequently observed in patients coinfected with human immunodeficiency virus (HIV) upon the initiation of antiretroviral therapy (ART). Herein, we present a unique case of a paradoxical reaction in a previously healthy 19-year-old female who started anti-tuberculosis treatment for disseminated tuberculosis. Four weeks after treatment initiation, she developed two new swollen masses in her left dorsum of the hand, accompanied by fever and new right submandibular painful lymphadenopathy, with worsening of the preexisting left lower neck lymph node. The patient underwent needle aspiration from her new skin abscess on the dorsum of her left hand, which revealed positive polymerase chain reaction (PCR) for *Mycobacterium tuberculosis*. Anti-tuberculosis treatment was continued, and the patient fully recovered. We described an unusual presentation of paradoxical reaction manifested by a skin abscess at a site distant from her primary disease in an immunocompetent TB patient, which demonstrated the importance of considering paradoxical reactions in HIV-negative patients who present with worsening signs and symptoms after initial improvement following treatment initiation.

## Introduction

A paradoxical reaction (PR) in patients diagnosed with tuberculosis (TB) was defined as clinical or radiological worsening of the preexisting disease while receiving anti-TB medications or the development of new lesions without a visible cause, not related to the usual course of the disease after the demonstration of an initial response to therapy [1]. These reactions are more commonly observed in patients coinfected with human immunodeficiency virus (HIV) after the initiation of antiretroviral therapy (ART) [2, 3]. Notably, the incidence of paradoxical reactions among HIV patients coinfected with TB following the initiation of ART has been estimated to be approximately 18%. However, paradoxical reactions can also occur in immunocompetent individuals [4]. The onset of PR can range from one week to several months after anti-TB initiation [5, 6]. Additionally, the PR manifestations that were described in the literature are derived mostly from case reports and case series and include a variety of manifestations, such as intestinal perforation, vertebral tuberculosis, tubercular liver abscess, retropharyngeal abscess, endobronchial obstruction, and other various syndromes [1, 7–10]. This phenomenon is usually diagnosed by excluding other causes of TB worsening, such as drug resistance, treatment failure, adherence issues, and secondary infections (Table 1) [11]. However, PR diagnosis can be delayed by the challenges surrounding TB culture.

**Table 1:** A clinical comparison between paradoxical reaction, drug resistance, and relapse in tuberculosis treatment

	Paradoxical reaction	Drug resistance	Relapse
Definition	• Clinical or radiological worsening of the preexisting disease while receiving anti-TB medications or the development of new lesions without a visible cause after the demonstration of an initial response to therapy [1]	• The occarance of TB disease due to a Mycobacterium tuberclusis complex strain resistant to any anti-TB medications [25]	• The occarance of a second or third episode of TB disease caused by re-emergence of the primary infection, as confirmed by genotypic tests [26]
Onset	Occurs within weeks of starting anti-Tb treatment [3]	Can occur anytime during treatment [25]	Can occur within months to years after initial treatment (typically within the first year after treatment) [26]
Risk factors	HIV co-infection (specifically low baseline CD4 count and shorter interval between starting anti-TB and ART) Extrapulmonary TB Disseminated TB [2]	Prior exposure to inappropriately dosed anti-TB or non-adherence during treatment of previous episode Traveling or living in an endemic area with high prevalence of drug-resistant TB Exposure to confirmed or suspected drug-resistant TB case [27]	Drug-resistant TB HIV co-infection with low baseline CD4 count Smoking Chronic lung disease Cavitary pulmonary TB Smear positive TB disease [28, 29]
Diagnosis	Diagnosed by excluding other causes of TB worsening (such as drug resistance, treatment failure, adherence issues, and secondary infections) [11]	Drug susceptibility testing [25]	Clinical history and symptom evaluation, whole-genome sequencing [26].
Management	Continue anti-TB treatment +/- anti-inflammatory drugs in severe cases [2, 4]	Requires change in treatment regimen [25]	Requires initiating anti-TB treatment [26]

We report a unique case of a 19-year-old immunocompetent otherwise healthy female who suffered from paradoxical worsening of disseminated TB during anti-TB treatment manifested as a skin abscess at a site distant from her primary disease.

## Case presentation

A 19-year-old Saudi female, previously healthy and a university student, presented to the emergency department with progressively increasing left-sided neck lymph nodes for 2 months. Two weeks before her presentation, her left-sided neck lymph node ruptured, and pus was draining.

She reported a history of dry cough, night sweats, fever, anorexia, and weight loss of 10 kg in the last six months. Her sister was diagnosed with pulmonary tuberculosis two years ago and received treatment. On examination, the patient was a tall and thin female (BMI = 19.05 kg/m2), and neck examination revealed an enlarged left cervical lymph node, including the draining sinus with purulent materials. The patient had a normal cardiopulmonary and abdominal examination.

**Fig. 1:**
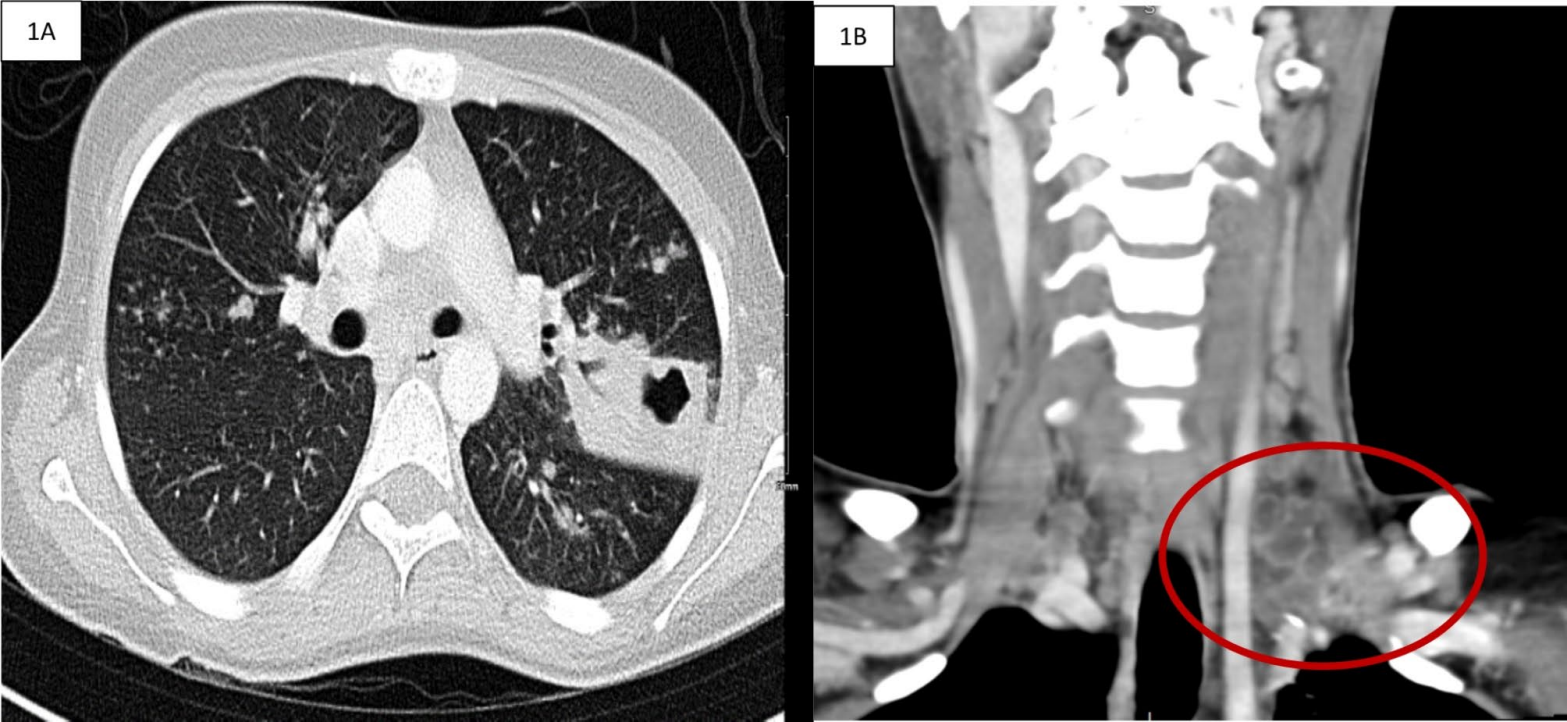
**A**: Axial enhanced computed tomography (CT) of the chest showed cavitating consolidation in the left upper lobe with few scattered satellite nodular opacities. Figure 1**B**: Coronal enhanced CT image of the neck showing multiple small and matted left supraclavicular lymphadenopathies

Her interferon-gamma release assay (IGRA) was positive, and her chest X-ray showed left upper lobe opacities. An enhanced computed tomography (CT) scan of the neck and chest revealed left supraclavicular necrotic lymph nodes and cavitating consolidation in the left upper lobe with few scattered satellite nodular opacities (Fig. 1). Her serology was negative for HIV. Her pretreatment white blood count (WBCs) was 6400–6600 cells/microliter, and her lymphocyte count ranged from 650 to 1200 cells/microliter, which is low (the normal range is 1500–4000 cells/microliter). In addition, She had iron deficiency anemia with an Hgb of 8.8 g/dl and hypoalbuminemia with an albumin level of 3.2 g/dl. Despite cavities on her chest CT, her dry cough made it difficult to conduct a sputum test for AFB/Xpert. Aspiration of purulent material from the lymph node sinus was negative for acid-fast bacilli smears; however, polymerase chain reaction (PCR) for *Mycobacterium tuberculosis* was positive without rifampin resistance according to the molecular rapid test (GeneXpert MTB/RIF method). After fifteen days of incubation, the culture of purulent material from cervical lymph nodes for acid-fast bacilli grew a *Mycobacterium tuberculosis* complex with the Mycobacteria Growth Incubator Tube (MGIT 960; Becton, Dickinson and Company, USA), which is sensitive to first-line anti-tuberculosis agents.

In accordance with international guidelines and recommendations, the patient was placed in airborne isolation during her hospital stay. She was started on standard weight-based anti-tuberculosis treatment comprising daily isoniazid 300 mg, rifampin 600 mg, ethambutol 800 mg, and pyrazinamide 1000 mg. Eleven days later, she complained of nausea and vomiting. Her laboratory investigations revealed elevated aspartate aminotransferase (AST) and alanine aminotransferase (ALT) levels, which were 304 and 154 IU/L, respectively. Other hepatic parameters were within the normal range. An enhanced computed tomography (CT) scan of the abdomen and pelvis showed no abnormalities. Anti-tuberculosis medications were withheld for a total of nine days, and we subsequently restarted ethambutol, moxifloxacin, rifampin, and isoniazid after her liver enzymes improved.

**Fig. 2:**
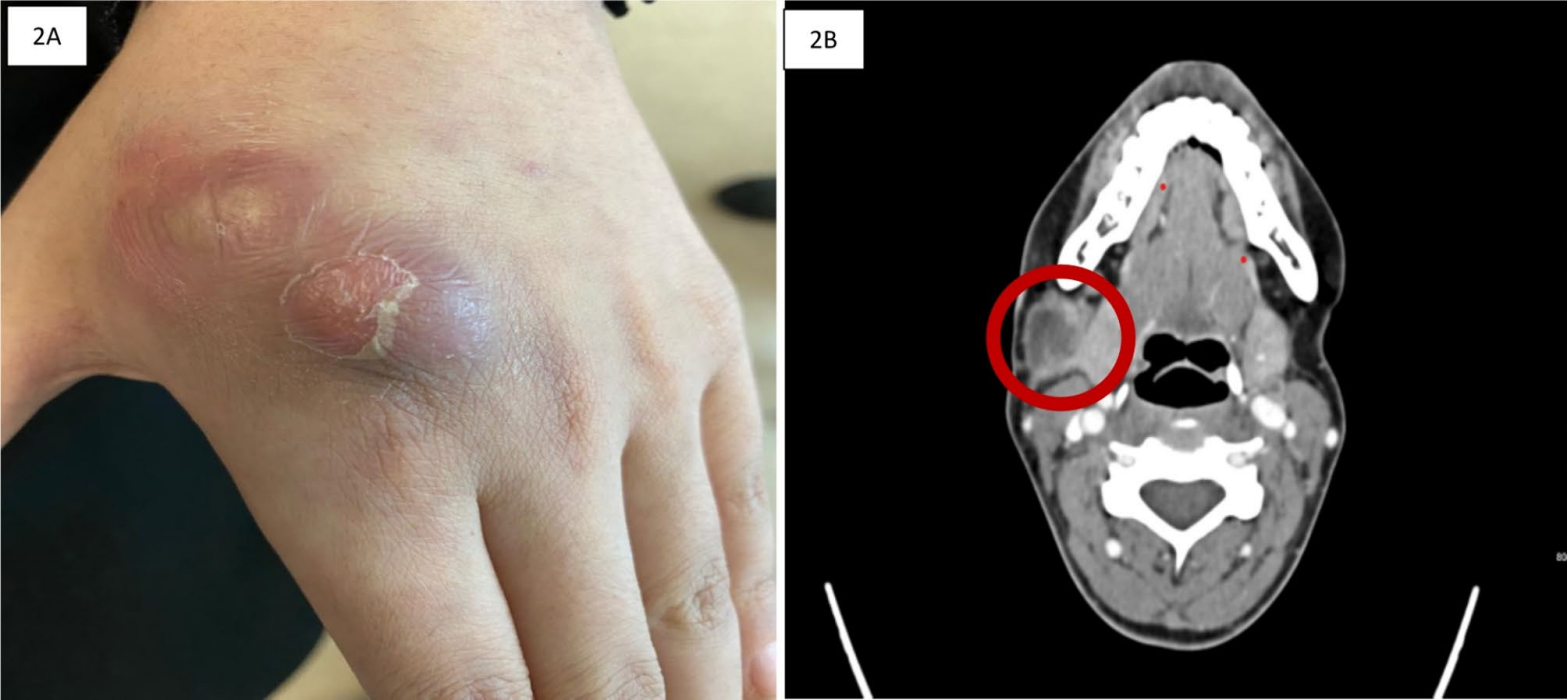
**A**: Appearance of a left-hand lesion after 4 weeks of restarting her on antituberculous treatment. Figure 2**B**: Enhanced CT scan of the neck four weeks after treatment with anti-TB agents showed an enlarged necrotic right submandibular lymph node

At the outpatient review four weeks later, she noticed new two swellings at the dorsum of her left hand. The swellings started to appear six days before her visit. Each swelling was around 3 centimeters. Physical examination demonstrated erythematous, fluctuant, painless swellings over the dorsal aspect of the left hand overlying the second metacarpal bone (Fig. 2A). There was no bony tenderness or limited motion of her hand or fingers. In addition, a left-hand X-ray was done which showed intact bone anatomy with no evidence of osteomyelitis. Needle aspiration yielded frank pus, which was sent for routine bacterial culture and sensitivity along with acid-fast bacilli and molecular testing. Bacterial culture and acid-fast bacilli smear and cultures were negative; however, polymerase chain reaction (PCR) for *Mycobacterium tuberculosis* (GeneXpert MTB/RIF method) was positive, and no rifampin resistance was detected. Two days later, she returned to the emergency department with fever, a temperature of 39 °C, new right submandibular painful lymphadenopathy, and worsening of the preexisting left lower neck lymph node with drainage (Fig. 2B). Aspiration was advised, which was declined by the patient.

Treatment with anti-TB agents was continued, and anti-pyritic agent (Paracetamol) was prescribed for fever. The patient showed clinical improvement with the resolution of her fever. Due to negative bacterial cultures and the patient’s improvement on antituberculosis medications, superadded infection was ruled out. Therefore, the most likely diagnosis was disseminated tuberculosis with a paradoxical reaction during antituberculosis treatment.

As for her immune status, fourth–generation HIV-1/2 immunoassay testing was done two times during her treatment. The first HIV testing was done at the initial presentation, and the second test was done two months later. Both tests came back negative. Additionally, her autoimmune profile was normal, and there was no history of recurrent infection, previous admissions, medication use, or family history of immunodeficiency.

She completed two months on four agent regimens, which included isoniazid, rifampin, ethambutol, and moxifloxacin, and then continued isoniazid, rifampin, and moxifloxacin for an additional 10 months with a very good clinical response. Compliance to treatment was ensured by regular follow-up during clinic visits and phone calls, inquiring about the patient’s medication intake, and ensuring adherence to her prescription, in addition to monitoring her improving clinical symptoms. A clinical follow-up 6 months after the completion of her therapy showed improvement without disease recurrence.

A positive polymerase chain reaction (PCR) results for *Mycobacterium tuberculosis* from her skin abscess at the dorsum of the left hand confirmed a paradoxical response to antitubercular treatment after excluding all other possible causes such as drug resistance, treatment failure, adherence issues, and secondary infections.

## Discussion

Paradoxical reactions are characterized by greater multisystem involvement and systemic inflammatory features, with pulmonary and lymph node involvement being more frequent features and abscesses and neurological features less frequently reported [2]. Risk factors for paradoxical reactions include extrapulmonary TB and disseminated TB [2]. According to a multivariate analysis by Namale et al., extrapulmonary TB was found to increase the risk of paradoxical reactions in HIV-infected adults by eightfold [2]. Furthermore, disseminated TB patients have a ninefold increased risk of paradoxical TB-IRIS [2]. The underlying mechanisms of paradoxical reactions in TB patients are not fully understood. However, an exaggerated immune response is believed to occur upon initiation of anti-TB therapy, leading to an inflammatory reaction [12]. In patients with HIV co-infection, immune reconstitution inflammatory syndrome (IRIS) can be an early complication of antiretroviral therapy (ART) that might result in paradoxical reactions in this population [13]. However, further investigations into the immunological mechanisms involved are needed to identify potential predictors and therapeutic targets for these reactions.

In our case, PCR from the patient’s skin abscess tested positive for *Mycobacterium tuberculosis* without rifampin resistance which further excludes drug resistance as a cause of the appearance of her new abscesses. PR reactions showing positive microbiological evidence of *Mycobacterium tuberculosis* have been reported previously in the literature [1, 7, 14, 15]. Garcia Vidal et al., reported four cases of PR to anti-TB treatment during infliximab therapy [14]. One of these cases was of a patient diagnosed with miliary TB who experienced a progressive enlargement of cervical lymph nodes two months after initiating anti-TB treatment [14]. Cultures of the tissue biopsy revealed pan-susceptible *Mycobacterium tuberculosis* [14]. In addition, Volpe-Chaves et al. reported a PR case of a patient with confirmed pulmonary and meningeal TB who developed a paravertebral abscess after two months of anti-TB treatment [1]. The patient had a positive PCR and culture for *Mycobacterium tuberculosis* from the drained material of the paravertebral abscess and achieved a positive outcome by continuing the anti-TB regimen [1].

Our patient’s BMI was on the lower end of the normal range (BMI = 19.05 kg/m2); otherwise, she was a healthy female who initially presented with disseminated disease. After four weeks of anti-TB treatment, she developed a paradoxical reaction. Notably, there is an association between malnutrition and TB incidence, emphasizing the bidirectional relationship between these two conditions [16–21]. The risk of progression from latent TB infection to active TB disease is increased by undernutrition, which further leads to weight loss [18, 21]. The association between malnutrition and TB is of particular importance in countries with high burdens where both conditions are prevalent.

In our case, through the continued use of anti-TB medications and close follow-up, the patient achieved favorable outcomes manifested by the spontaneous resolution of hand abscesses without the use of anti-inflammatory agents or surgical intervention. However, in a case reported by Gao et al., a 48-year-old man with dermatomyositis developed multiple subcutaneous tuberculous abscesses in his limbs, including one that developed during anti-TB treatment as a paradoxical reaction [22]. The patient underwent routine anti-TB therapy, local puncture drainage, and surgical resection, which resulted in a favorable outcome [22].

The management of paradoxical reactions in TB depends on the severity of the symptoms and the organ system involved. In some cases, no specific treatment other than continuing anti-TB therapy is needed, and spontaneous resolution of the patient’s symptoms might occur without intervention [4]. However, in more severe cases, additional interventions may be necessary, including anti-inflammatory agents such as corticosteroids and nonsteroidal anti-inflammatory drugs (NSAIDs) [2, 23]. Corticosteroids have been used to treat paradoxical reactions in TB patients, especially in those with central nervous system involvement or protracted usage of corticosteroids [23]. In the systematic review and meta-analysis of PRs in HIV-infected adults by Namale et al., corticosteroids were prescribed more frequently than NSAIDs for the treatment of paradoxical reactions in TB patients (38% vs. 28%, respectively) [2]. In addition, surgical interventions, such as drainage or resection, may be required for abscesses or perforations [22, 24]. The management of paradoxical reactions in TB patients should be individualized based on the patient’s clinical presentation and response to treatment.

In conclusion, we described an unusual presentation of paradoxical reaction manifested by a skin abscess at a site distant from her primary disease in an immunocompetent TB patient after an initial improvement following anti-TB treatment. Through continued anti-TB treatment and close follow-up, the patient was successfully cured without surgical intervention. Paradoxical reactions should be considered in patients who present with worsening signs and symptoms after initial improvement following anti-TB therapy, especially those with risk factors such as HIV coinfection, disseminated TB, and extrapulmonary TB, after excluding other causes. However, further research is needed to understand the immunological mechanisms underlying these reactions and to identify potential prognostic factors and therapeutic targets.

## Data Availability

No datasets were generated or analysed during the current study.
